# Ultrafast creation of a light-induced semimetallic state in strongly excited 1T-TiSe_2_

**DOI:** 10.1126/sciadv.adl4481

**Published:** 2024-05-10

**Authors:** Maximilian Huber, Yi Lin, Giovanni Marini, Luca Moreschini, Chris Jozwiak, Aaron Bostwick, Matteo Calandra, Alessandra Lanzara

**Affiliations:** ^1^ Materials Science Division, Lawrence Berkeley National Laboratory, Berkeley, CA 94720, USA.; ^2^ Department of Physics and Astronomy, University of Alabama, Tuscaloosa, AL 35487, USA.; ^3^ Graphene Labs, Fondazione Istituto Italiano di Tecnologia, I-16163 Genova, Italy.; ^4^ Department of Physics, University of Trento, 38123 Povo, Italy.; ^5^ Advanced Light Source, Lawrence Berkeley National Laboratory, Berkeley, CA 94720, USA.; ^6^ Sorbonne Universite, CNRS, Institut des Nanosciences de Paris, F-75252 Paris, France.; ^7^ Physics Department, University of California, Berkeley, Berkeley, CA 94720, USA.; ^8^ Kavli Energy NanoScience Institute, Berkeley, CA 94720, USA.

## Abstract

Screening, a ubiquitous phenomenon associated with the shielding of electric fields by surrounding charges, has been widely adopted as a means to modify a material’s properties. While most studies have relied on static changes of screening through doping or gating thus far, here we demonstrate that screening can also drive the onset of distinct quantum states on the ultrafast timescale. By using time- and angle-resolved photoemission spectroscopy, we show that intense optical excitation can drive 1T-TiSe_2_, a prototypical charge density wave material, almost instantly from a gapped into a semimetallic state. By systematically comparing changes in band structure over time and excitation strength with theoretical calculations, we find that the appearance of this state is likely caused by a dramatic reduction of the screening length. In summary, this work showcases how optical excitation enables the screening-driven design of a nonequilibrium semimetallic phase in TiSe_2_, possibly providing a general pathway into highly screened phases in other strongly correlated materials.

## Teaser

Optical excitation can drastically modify the electronic structure of TiSe_2_ and drive it to a nonequilibrium semimetallic state.

## INTRODUCTION

Understanding the effect of screening is a prerequisite step for understanding and manipulating many-body interactions in quantum materials in and out of equilibrium. Most previous studies have focused on manipulating screening properties by tuning the carrier density [doping (*1*–*5*) or electrostatic gating (*6*–*8*)]. More recently, sample thickness has been used as another control knob, for instance, in transition metal dichalcogenides, where the reduced dimensionality in the monolayer limit leads to reduced screening and thus to drastically enhanced exciton binding energies (*9*). Photodoping is a particularly effective tool to manipulate the electronic properties of a material via screening compared to doping with external carriers in equilibrium, as the optical excitation leads to the quasi-instantaneous creation of hot electrons and holes simultaneously. The photoinduced renormalization of bandgap or many-body interactions has been reported in, among others, semiconductors (*10*, *11*), superconductors (*12*–*14*), and topological insulators (*15*). In extreme cases, such abrupt changes can drive materials into nonequilibrium phases that cannot be obtained under equilibrium conditions (*16*–*20*). The sensitivity of the prototypical charge density wave (CDW) material 1T-TiSe_2_ to electronic interactions (*21*, *22*) makes it an ideal system to study the effect of photoinduced screening-driven states beyond equilibrium. In particular, the strong Coulomb repulsion due to the localized nature of the Ti_3*d*_ orbitals makes the theoretical description of TiSe_2_ intricate even at equilibrium and, as a result, (semi)local functionals are not able to accurately describe the electronic structure of the normal and CDW state (*21*). While the introduction of the Hubbard *U* can, in principle, cure this well-known delocalization error, leading to very accurate electronic structures, it fails to reproduce the structural properties and the CDW instability (*23*). On the other hand, the use of the computationally more expensive hybrid functionals allows us to calculate both the electronic structure and phonon spectrum in accordance with experimental data (*21*, *22*). Studying TiSe_2_ under highly out-of-equilibrium conditions is furthermore of interest in view of the recent report of a metastable metallic phase (*20*) and to shed light into one of the ongoing debates in the field, i.e., how its electronic structure evolves from the CDW to the normal state (*24*–*29*). In this regard, angle-resolved photoemission spectroscopy (ARPES) is the ideal tool to study screening effects in and out of equilibrium (*1*, *2*, *30*), as the simultaneous momentum and energy resolution allows the direct visualization of the evolution of the band structure upon photoexcitation as well as the detection of changes of many-body interactions (*31*, *32*), information not easily directly accessible through other techniques. Our results reveal an ultrafast transition from the gapped CDW phase into a semimetallic state, which happens within the temporal resolution of our experiment, i.e., in under 55 fs. Careful analysis as a function of excitation density shows that the band structure gets increasingly renormalized with increasing pump fluence, with the valence band gradually opening up until it shows an almost linear dispersion crossing the Fermi level. With the band structure being clearly different than both the equilibrium high- and low-temperature state, we use density functional theory (DFT) simulations to show a remarkable similarity between the experimental data and calculated quasiparticle band structures for the CDW state with screened electron-electron interactions and substantially reduced lattice order. In summary, this work demonstrates how intense laser pulses can drive nonequilibrium states of matter where electron-electron and possibly electron-lattice interactions are highly suppressed.

## RESULTS

**Fig. 1.**
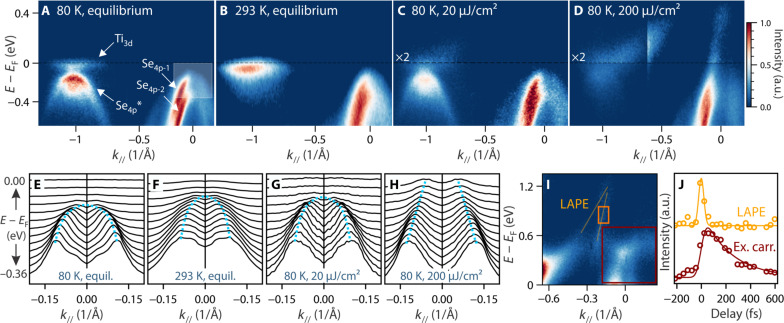
Overview of the electronic band structure of TiSe_2_ in equilibrium and after excitation. (**A** and **B**) ARPES spectra of the *L* and *A* point in equilibrium at 80 K (A) and at room temperature (B), respectively. (**C** and **D**) ARPES spectra after excitation with 780-nm pump pulses with 20 μJ/cm^2^ (C) and 160 μJ/cm^2^ (*L* point) and 200 μJ/cm^2^ (*A* point) (D), respectively, after 80 fs. Spectra at *L* and *A* points are normalized independently. For clarity, excited states in (C) and (D) are shown with higher intensity and after subtraction of an exponential background above the Fermi level. The Fermi level is indicated by the dashed black line. (**E** to **H**) Stacked MDCs taken between −0.36 and 0 eV with focus on the *A* point corresponding to spectra in (A) to (D). Band dispersion is schematically shown as a guide to the eye by the dashed blue lines. The region from which MDCs are extracted is shown in (A) by the white shaded area. (**I**) APRES image plot at the *A* point after excitation with 180 μJ/cm^2^ around 0 fs. Bands due to the LAPE effect are marked in orange. For clarity, an exponential background was subtracted. (**J**) Dynamics of LAPE (orange) and excited carriers (red) after excitation with 180 μJ/cm^2^. Integration regions are indicated in (I). Solid lines are fits to the data points (Gaussian function for the LAPE, exponential decay for excited carriers).

Figure 1 shows the evolution of the electronic band structure of the CDW phase following an optical excitation by a near-infrared pump pulse of 1.6 eV (780 nm). Data are taken with a probe energy of 22.3 eV. See Methods for more details of the extreme-ultraviolet (XUV) time-resolved (tr)–ARPES experiment and setup. Figure 1A shows the experimental equilibrium quasiparticle band structure measured at 80 K, i.e., below the CDW transition temperature [*T*_CDW_ *~*200 K (*33*)], in the *k_z_* plane close to the *A*-*L* direction (*24*, *34*, *35*) in the bulk Brillouin zone (BZ) notation. The onset of the CDW state in TiSe_2_ is accompanied by a periodic lattice distortion (PLD), which leads to a doubling of the unit cell size into a (2 *×* 2 *×* 2) superstructure (i.e., a folding of the BZ) from the typical (1 *×* 1 *×* 1) structure in the normal state (*24*, *33*). As a consequence of CDW/PLD formation, the Se_4*p*_ valence band from the *A* point gets folded onto the *L* point (Se_4p_* band), where a gap between the Se_4p_* band and the Ti_3*d*_ conduction band opens up. The high momentum and energy resolution of this experiment allow us to directly visualize those spectral fingerprints of the CDW state. Furthermore, the spin-orbit splitting of the valence band (Se_4*p*-1_ and Se_4*p*-2_) is clearly resolved. The experimental band structure of the CDW state is, overall, in very good agreement with our band structure calculations and previous reports (*25*, *27*, *34*). At room temperature (Fig. 1B), i.e., above the CDW transition temperature, the folded band at *L* disappears almost completely, and the valence band at *A* shifts slightly upward (by *~*50 meV), still leaving an *~*80-meV indirect gap between the valence and conduction band, in accordance with previous reports (*24*, *34*, *36*, *37*). Figure 1 (C and D) shows the evolution of the CDW state following pump excitation, in the weakly and strongly excited regimes, respectively. In the weakly excited regime (Fig. 1C), we observe a reduction of the intensity of the backfolded band, a slight decrease of the CDW gap, and the occupation of the Ti_3*d*_ band by hot electrons, in line with previous reports (*27*, *37**–**39*). Unexpectedly, when the system is excited with stronger laser pulses (Fig. 1D), a completely different feature appears at the *A* point. The valence band opens up with its dispersion changing drastically from the parabolic to a linearly dispersive band shape, which extends all the way above the Fermi level. Previous trARPES studies (*20*, *27*, *39**–**42*) have not reported such a state, nor have equilibrium experiments at a high temperature or with doping (*24*, *25*). To further visualize the light-induced dispersion changes, in Fig. 1 (E to H), we show the momentum distribution curves (MDCs) (curves at constant energy as a function of momentum) with focus on the valence band at the *A* point. Weak excitation or raising the temperature in equilibrium (compare Fig. 1, F and G, with Fig. 1E) induces a small upshift of the Se_4*p*_ bands and slightly affects the effective mass in comparison to the CDW equilibrium phase in Fig. 1E. Despite the minor changes, the overall shape of the band remains parabolic in both cases, with the maximum below the Fermi level. For further details on the low-excitation regime, we refer to our previous work (*37*, *38*), where we have extensively studied photoinduced changes of both the dispersion and bandgap. In drastic contrast, in the strong excitation regime, the low-temperature spectrum is remarkably different from the equilibrium low- and high-temperature spectra (compare Fig. 1, E to H). Specifically, the data show the opening of the Se_4*p*-1_ band with consequent crossing of the Fermi level and an evolution from a parabolic to an almost linear dispersion.

After showing the existence of a light-induced semimetallic state after strong excitation, we want to turn the attention to the ultrafast timescale on which it emerges. Figure 1I shows the two-dimensional image plot of the excited states above the Fermi level taken with very high statistics. The excellent energy resolution allows us to resolve, in addition to the linear excited state, the two Se_4*p*–1_ and Se_4*p−*2_ replica bands due to the laser-assisted photoelectric effect (LAPE, indicated by orange lines). The LAPE effect is a dressing of the photoemitted electron with the pump laser field (*43*), and, hence, it can only occur within the temporal width of the pump pulse. Therefore, its coexistence with the semimetallic state suggests that the transition into this regime happens almost instantly after excitation within the temporal resolution of the experiment. Figure 1J reports the dynamics of the excited carriers above the *A* point (integration region shown by the red box in Fig. 1I). The orange curve shows the dynamics of the LAPE effect, which, as mentioned above, by its own nature occurs at *t* = 0 and is thus often used in pump and probe ARPES experiments as reference for the temporal overlap. Fitting the curve with a Gaussian gives a full width at half maximum of 55 *±* 7 fs, which indicates the temporal resolution of the experiment. In contrast to the LAPE effect, the excited carrier population (red) reaches its maximum shortly after excitation at approximately *t* = 60 fs, indicating that the observed semimetallic state is not an intrinsic effect due to the photoemission process. By fitting the data with an exponential function, convoluted with a Gaussian (to account for the experimental time resolution), we extract a decay time of 210 *±* 17 fs. This fast decay of excited carriers is a further sign of a light-induced metallization of the sample and indicates that there are no larger gaps. In the presence of a pronounced gap, we would expect to observe a bottleneck in the carrier relaxation as it is the case for other gapped systems (*44*, *45*).

**Fig. 2.**
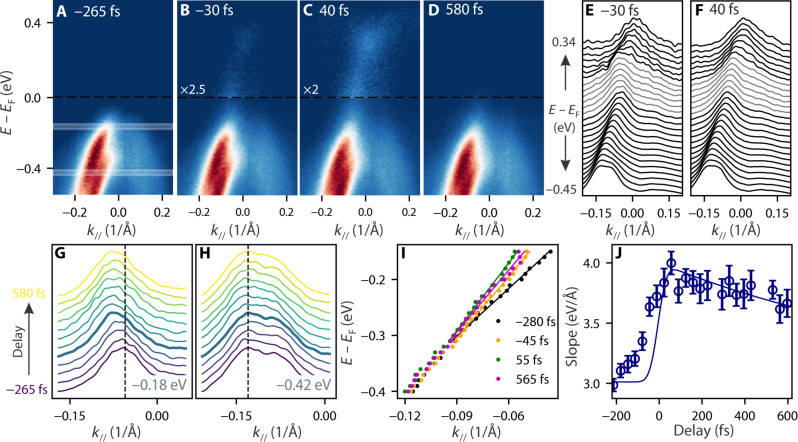
Time dependence. (**A** to **D**) ARPES image plots at delay values of −265 fs (A), −30 fs (B), 40 fs (C), and 580 fs (D) after excitation with 180 μJ/cm^2^. All spectra were aligned in energy to the side of the Se_4*p*-1_ band at −0.12 Å^−1^. For better clarity, the shown plots are averaged over 66 fs (i.e., two delay values). (**E** and **F**) Stacked MDCs corresponding to the spectra in (B) and (C), respectively. For clarity, each MDC slice is normalized to the same height. The energy region in which the band is less dispersive is indicated by the gray lines. (**G** and **H**) MDCs at different delay values integrated around −0.18 eV (G) and −0.42 eV (H), respectively. Integration regions are schematically shown in (A). For enhanced clarity, the MDCs were averaged over 66 fs. (**I**) Extracted dispersion of the Se_4*p*-1_ band for different delays with focus on below *E*_F_. Solid lines are linear fits to the data points. (**J**) Dynamical behavior of the slope extracted from linear fits to the valence band dispersion.

**Fig. 3.**
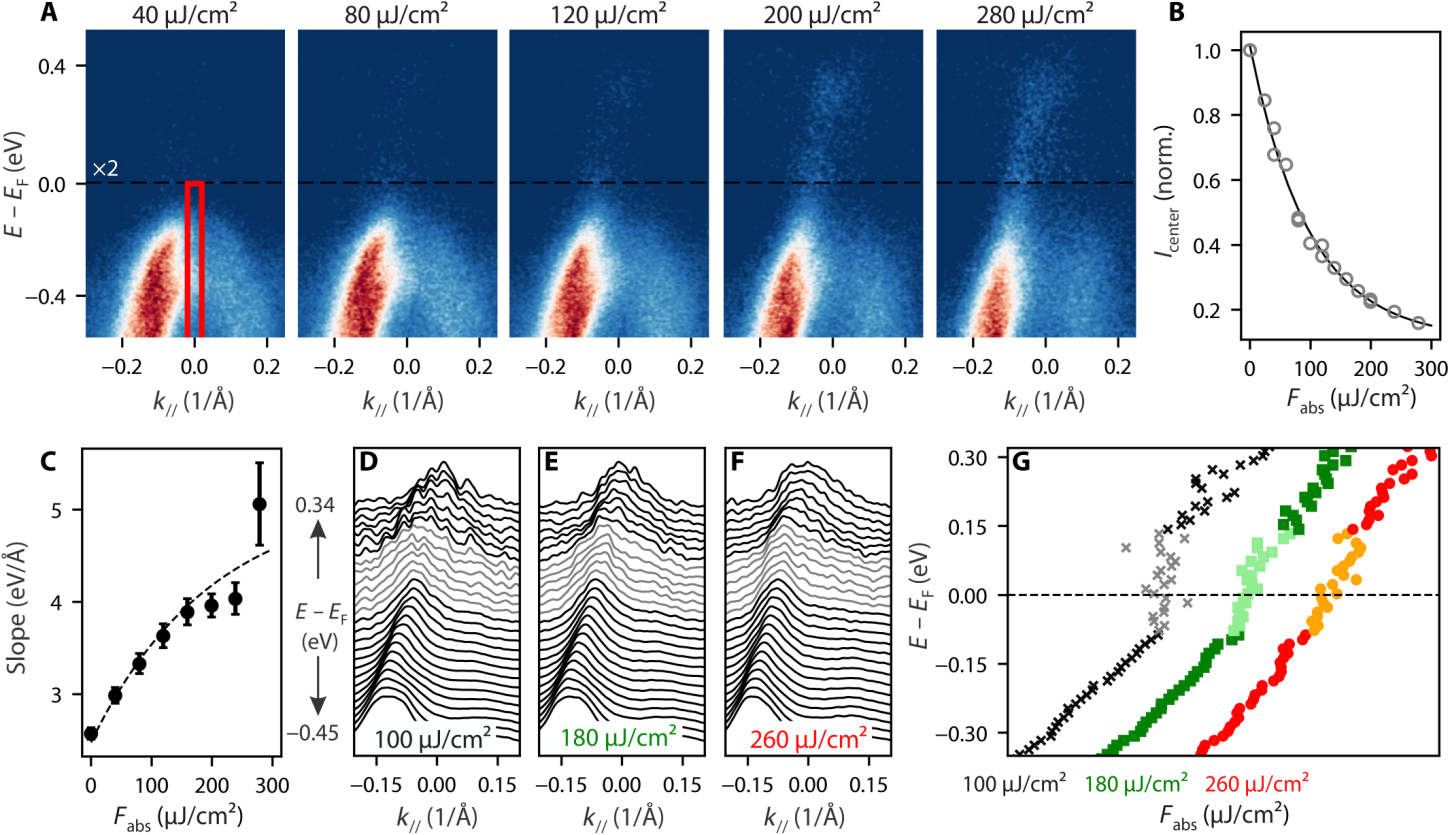
Fluence dependence. (**A**) ARPES plot at a delay of 85 fs after excitation with various fluences. (**B**) Integrated intensity at the *A* point. Integration region is shown by the red box in (A). (**C**) Extracted slope of the fitted Se_4*p*-1_ dispersion for different fluence values. (**D** to **F**) Stacked normalized MDCs for different fluences. For better signal-to-noise ratio, the shown MDCs are taken from spectra averaged over two fluence values, i.e., over a window of 80 μJ/cm^2^ after correcting for band shifts. The region in which the band is less dispersive is indicated in gray. (**G**) Extracted dispersion of the Se_4*p*-1_ band extracted from MDCs shown in (D) to (F).

Having shown the ultrafast and intrinsic nature of the optically driven semimetallic state, we now proceed to a quantitative study of its evolution as a function of time (Fig. 2) and pump fluence (Fig. 3). To compensate for rigid energy shifts, for instance, due to space charge effects, all spectra that are analyzed in the following are aligned in energy with respect to the side of the Se_4*p*-1_ valence bands (see note S1 for more details). Figure 2 (A to D) shows the ARPES spectra for different delay times, where the transition from the gapped into the semimetallic state can be clearly followed. Because of the finite width of the laser pulses, carriers get excited even before the maximum of pump and probe pulse overlap at *t* = 0 fs. Already at very early delay times (−30 fs), we can observe excited carriers that populate a seemingly linearly dispersing band above the Fermi level. A closer look reveals that there is a discontinuity between the dispersion of the bands populated by hot electrons above the Fermi level and the valence band below, whereas at later times (*t* = 40 fs; Fig. 2C), both bands are connected. To analyze these subtle differences in more detail, in Fig. 2 (E and F), we plot stacked MDCs, corresponding to the spectra shown in Fig. 2 (B and C, respectively). To compensate for the large intensity difference of states above and below *E*_F_, we normalized each MDC slice individually to the same height. At −30 fs (Fig. 2E), one can clearly identify a region of about *~*200 meV marked in gray where the MDC peaks do not disperse. The presence of nondispersive MDCs in ARPES spectra hints toward an energy gap (*46*, *47*), where, in this particular case, the persistence of peaks in the gap region is caused by broadening effects due to the highly out-of-equilibrium nature of the experiment and the large intensity difference between the original valence band and states above the Fermi level only populated by excited carriers. In contrast, at a slightly later delay time (Fig. 2F), the MDC peaks show a seemingly linear trend over the entire energy range, indicating an almost continuous, linearly dispersing band with no or only a small gap. To better understand the opening dynamics of the valence band, in Fig. 2 (G and H), we show the MDC spectra near the top of the valence band around −0.18 eV and at lower energy −0.42 eV. The spectra are integrated over an energy window of 20 meV. Note again that to analyze changes in the band dispersion free of rigid band shifts, the spectra were aligned in energy before extracting the MDCs (note S1). In the vicinity of the Fermi level (Fig. 2G), the MDC peaks show a pronounced shift away from the *A* point, with the maximum shift observed at around *t* = 0 fs (thick blue line). In contrast, the MDCs at lower binding energies (Fig. 2H) barely show any shift, suggesting that the changes in the proximity of the Fermi level are in fact due to an opening of the Se_4*p*-1_ band, eventually leading to the almost linear dispersion relation almost instantly after excitation. To quantify those changes, in Fig. 2I, we show the Se_4*p*-1_ dispersion extracted from standard Lorentzian fitting (*31*, *32*) of the MDC spectra of the upper part of the valence band (see note S2). We note that as shown in Fig. 1, the band structures between equilibrium and semimetallic state are drastically different. While energy distribution curves are better in capturing the parabolic shape in equilibrium, MDCs are better suited to describe the dispersion of the linear bands of the semimetallic state. As we are interested in the timescale of the transformation into the semimetallic state, we hence consistently extract the dispersion for all delay values using MDCs. The timescale associated with the valence band opening is shown in Fig. 2J, where the change of the band velocity (slope) obtained through a linear fitting of the top of the valence band is shown as a function of delay time (fits for selected delay times are shown in solid lines Fig. 2I). The maximum change occurs around *t* = 0 fs and is followed by a slow recovery. Within 600 fs, the slope has still not fully recovered back to its equilibrium value, which is in contrast to the excited carriers that decay back to equilibrium within a short timescale (Fig. 1J). Further discussion on the possible origin of this slow recovery is presented later.

After having shown the light-induced ultrafast temporal evolution from the gapped CDW into the semimetallic state, we now proceed to analyze the transition as a function of fluence. Figure 3A shows ARPES image plots at a delay of 85 fs for different excitation densities. At lower fluences, the valence band shows the standard parabolic dispersion, typical of the CDW phase. As the excitation density increases, the Se_4*p*-1_ band opens up, and a linear dispersion relation develops, which, as discussed above, signals the onset of a semimetallic phase. To quantify the photoinduced changes, we plot the ratio of the integrated intensity at the center of the *A* point with respect to the equilibrium value as a function of fluence (Fig. 3B). The integration region is shown by the red box in Fig. 3A. After a fast initial decrease for low-excitation densities, the quench of the intensity starts to saturate at higher fluences, which might be caused by a saturation of the optical absorption (*48*). As multiple factors like band depopulation or band renormalization can cause the observed decrease of intensity, we report the fluence dependence of the Se_4*p*-1_ band velocity as a more direct measure of the opening of the valence band in Fig. 3C. The velocity (slope) is extracted in the same way as discussed in the previous figure (Fig. 2). The trend can be described by an exponential function, with a fast increase of the slope at low fluences followed by a slowing down at higher fluences. For a more detailed analysis, in Fig. 3 (D to F), we again plot normalized stacked MDCs in the same way as in Fig. 2 (E and F) for different fluences. By fitting the MDCs with the standard Lorentzian fitting method (see note S2) (*31*, *32*), we extract the Se_4*p*-1_ dispersion shown in Fig. 3G. Similar to the data point at very early delay time (Fig. 2A), for weak excitation density, we can identify a large region around the Fermi level where the MDC peaks do not disperse, indicating a pronounced gap. With increasing fluence, one can observe how this region gradually becomes more dispersive until finally at high fluences the band above and below merge into one continuous band. For the highest fluence studied (280 μJ/cm^2^), we estimate an indirect band overlap between the bottom of the Ti_3*d*_ conduction band at the *L* point and the top of the valence band at the *A* point of about ~350 meV (see note S3). This value is drastically different from that reported in previous equilibrium studies at room temperature, where usually an indirect gap of *~*75 meV (*24*, *34*, *36*) is observed and further highlights the nonequilibrium nature of the light-induced semimetallic state.

In summary, the data shown so far unambiguously prove the existence of a linearly dispersing, photoinduced semimetallic state in TiSe_2_, which, to the best of our knowledge, has not been observed so far. This might be the result of photoemission matrix element effects due to the use of different polarizations (*39*) or photon energies (*20*, *27*, *40**–**42*) of the probe beam in previous work. As shown in note S4, the visibility of the Se_4*p*-1_ band forming the semimetallic state is strongly dependent on the polarization of the probe beam. Moreover, we find that extrinsic factors such as sample and cleave quality tend to strongly suppress the intensity of the Se_4*p*-1_ band. The light-induced semimetallic state is remarkably different from both the equilibrium low- and high-temperature state [Fig. 1, A and B; (*24*, *34*, *36*)]. Even increasing the temperature far above room temperature (note S5) or heavy doping (*25*, *49*–*52*) does not show the drastic renormalization of the valence band observed in this work. Since photon energies and polarization are easily tunable in synchrotron experiments, and the resolution is generally higher than for XUV tr-ARPES experiments, we take this as further support of the nonequilibrium nature of the light-induced semimetallic state. Figure 1D seems to suggest that there is a regime in which the band structure can become semimetallic, while there is still a finite PLD (indicated by residual intensity of the folded Se_4p_* band), a scenario that is impossible for both a Peierls and an excitonic insulator–driven CDW phase under equilibrium conditions (*26*, *53*).

**Fig. 4.**
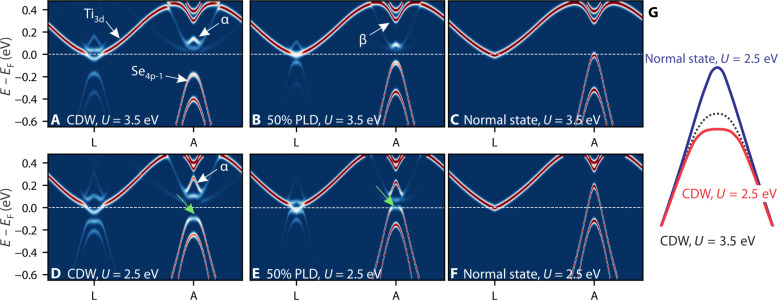
DFT+ U calculations. Calculated single-particle band structures for different lattice distortions and Hubbard *U* terms. Upper row (**A** to **C**) shows the band structure as a function of PLD for* U *= 3.5eV, bottom row (**D** to **F**) shows the band structure as a function of PLD for *U *= 2.5eV.

To get a better insight into the origin of this light-induced semimetallic state, we perform first-principles DFT calculations. Femtoseconds after laser irradiation, we expect a substantial enhancement of the electronic screening of the Coulomb interaction due to the higher density of nonequilibrium free electronic carriers. To qualitatively capture this effect in TiSe_2_, we describe the electronic properties of both the normal and CDW phases within the DFT + *U* formulation (*23*, *54*). Within the DFT + *U* approach, an enhanced screening due to free carriers naturally leads to an effective renormalization of the Hubbard *U* value (*55*–*57*), which represents the short-range Coulomb repulsion. Besides screening of electron-electron interactions, diffraction experiments also indicate a fast suppression of PLD order after optical excitation (*41*, *58*). Thus, to qualitatively understand the nonequilibrium behavior of photoexcited TiSe_2_, we systematically vary the strength of lattice distortion and electron-electron interactions. This is shown in Fig. 4, where the electronic band structure for *U* = 3*.*5 eV (the equilibrium *U* value) and *U* = 2*.*5 eV (the reduced *U* value) are compared (see note S6 for further technical details on the theoretical spectra). As the system is initially found in the CDW phase, we start by analyzing the electronic structure of the CDW phase unfolded onto the Brillouin zone of the TiSe_2_ normal phase in Fig. 4A, where the main features are consistent with the present experiment (compare to Fig. 1A) and previous literature (*23*). In the top row (Fig. 4, A to C), we show the evolution of the band structure with decreasing PLD, which resembles the equilibrium phase transition with increasing temperature as studied in literature. The main changes at the *A* point are an upward shift of the valence band with respect to the CDW state (Fig. 4A) accompanied by a simultaneous downshift of the hole-like band (α) above the Fermi level, where both bands eventually merge into each other into a semimetallic band structure in the normal state with no PLD. At the *L* point, the main change is the disappearance of the backfolded Se band and the closure of the CDW gap. In the bottom row, we consider the effect of the enhanced screening (reduced *U* value) on the spectra. These calculations model the (possible) partial nonthermal melting of the charge-density wave order. For all lattice orders, we observe substantially modified quasiparticle dispersion, with important effects on the spectral intensity of both the valence and conduction bands in proximity of the *A* point of the BZ. In particular, in the CDW state with decreased *U*, the top of the Se_4*p*-1_ valence band, originally parabolic, becomes flatter and loses intensity (see green arrow), while the hole-like α band above the Fermi level acquires additional spectral weight. At the same time, the Se_4*p*-1_ band shifts upward, and the CDW gap at the *L* point becomes smaller. Moreover, the decrease in *U* causes the α band to disperse across a larger energy region (with the top of the band now lying around 300 meV above *E*_F_) while still remaining clearly separated from the valence band by a gap *>*200 meV. With decreasing PLD (Fig. 4E), the gap at the *A* point progressively vanishes, and the upper valence band reaches the Fermi level, together with a further decrease of the intensity of the top of the Se band at the *A* point (green arrow). An interesting point is that while for the CDW state with unperturbed lattice, the system remains semiconducting both with full and reduced Hubbard *U* (Fig. 4, A and D), for 50% PLD, a decrease in Hubbard *U* changes the electronic properties from semiconductor (Fig. 4B) to semimetal (Fig. 4E). Last, for the normal state with decreased *U*, where the PLD is fully quenched into the normal 1 *×* 1 *×* 1 lattice order (Fig. 4F), we observe the merging of the hole-like feature above the Fermi level (α) with the valence band below, following the full opening of the valence band as well as a sharpening of the top. Here, the overlap between valence band at *A* and conduction band at *L* is drastically enhanced compared to the normal state with *U* = 3.5 eV (Fig. 4C). To better visualize the changes in band structure, in Fig. 4G, we plot the calculated Se_4*p*-1_ valence band dispersion for different scenarios on top of each other and aligned in energy to the side of the band, identical to the alignment of our experimental data in Figs. 2 and 3. The comparison highlights how in the CDW state a change in *U* only leads to a flattening of the band, whereas a decrease in PLD is necessary to cause an opening of the top of the Se band.

Comparing these calculations with the experimental results, we find an excellent agreement between the band structures with reduced Hubbard *U* (Fig. 4, D to F) and the ARPES spectra taken after optical excitation. For very early delays (−30 fs; Fig. 2, B and E) and in the intermediate- to low-fluence regime (Fig. 3, D and E), we observe states where the top of the Se_4*p*-1_ valence band partially opens up, while excited carriers populate a linearly dispersing band above the Fermi level, with both bands being separated by a large energy gap on the order of *~*200 meV. These spectra resemble the calculated band structures with enhanced Coulomb screening (reduced *U*) and slightly reduced PLD (Fig. 4, D and E). With increasing fluence, this gap closes, and we observe the opening of the parabolic valence band and its evolution into a linearly dispersing band with a maximum far above the Fermi level at the *A* point. This is similar to the calculated evolution of the band structure with decreasing PLD into the normal state with reduced *U* in Fig. 4 (E and F). We also note that other general features of the calculations like the extension of the conduction band from the *L* point into the electron pocket at the *A* point (labeled β in Fig. 4) are reproduced as well (Fig. 1I). The excellent agreement of the calculated band structures with reduced *U* and our experimental data suggest that the observed semimetallic states are driven by screening effects. This is further supported by the ultrafast timescale on which the semimetallic state appears (Fig. 1, I and J and Fig. 2) and the correspondence of the opening dynamics with the build-up time of the excited carriers (compare Fig. 2J with Fig. 1J). In this context, the slow recovery of the slope shown in Fig. 2J, contrasting the fast decay of excited carriers (Fig. 1J), might seem somewhat unususal. Our calculations can account for this trend under the hypothesis in which the system relaxes from the normal state with reduced *U* into a state that is more similar to the equilibrium normal state (i.e., with *U ~* 3.5 eV) after 600 fs (*59*). This could eventually be also compatible with the light-induced metastable state recently reported in (*20*), for which lifetimes longer than several picoseconds were observed.

We finally want to emphasize that according to the calculations for the opening of the top valence band, a quench of PLD order is necessary (Fig. 4G), and for a complete transition into the semimetallic state as observed in the high-fluence regime, the lattice needs to transition completely into the normal state order. Since ARPES is only directly sensitive to the electronic order, it is ambiguous whether there is actually a quench of the PLD on such a fast timescale or if the semimetallic state is observed because the electronic subsystem becomes decoupled from the lattice order due to the strong optical excitation. Indications of the electronic order reacting faster to optical excitation have been observed in multiple experiments (*60*–*62*); in particular, for the case of TiSe_2_, it has been reported that at very low temperatures, lattice order can persist while the electronic order is fully quenched (*28*). In addition, recent work has shown that optical excitation leads to a weakening of the electron-phonon coupling strength (*63*, *64*) in TiSe_2_.

## DISCUSSION

In conclusion, we have shown how intense light pulses can modify the band structure of TiSe_2_ almost instantly via screening, transitioning it into a nonequilibrium semimetallic state. This result emphasizes how optical excitation can be used as a knob to tune electron-electron and electron-lattice interactions in solids while driving phases with drastically different properties unreachable in equilibrium.

## METHODS

tr-ARPES measurements were conducted at the Lawrence-Berkeley National Laboratory with 22.3-eV XUV femtosecond pulses. Photoelectrons are detected with a hemispherical electron analyzer (Scienta R4000). The light source is a cryo-cooled regenerative amplifier (KMLabs Wyvern 500) seeded by the output of a homebuilt, 76-MHz Ti:sapphire oscillator, which is pumped by 4.5 W from a green solid-state laser (Lighthouse Photonics Sprout). The amplifier stage, in turn, is pumped by two green, nanosecond-pulsed Nd:YVO_4_ lasers (Photonics Industries DS20HE). The XUV is created after second harmonic generation by tightly focusing 390-nm pulses into Kr gas. A detailed description of the setup can be found in (*65*). For the experiment, a pump wavelength of 780 nm with a repetition rate of 25 kHz was used with a total energy resolution of *~*75 meV and a temporal resolution of about 55 fs.

## Supplementary Materials

### This PDF file includes:

Supplementary Text Figs. S1 to S7 References
