# Supplementary material for: Ultrafast creation of a light-induced semimetallic state in strongly excited 1T-TiSe2
Source: Sci Adv. 2024 May 10;10(19):eadl4481. doi: 10.1126/sciadv.adl4481 (PMC11086600; doi:10.1126/sciadv.adl4481)
Supplement: Supplementary file 1 — Supplementary Text Figs. S1 to S7 References [file sciadv.adl4481_sm.pdf]

Science Advances
AAAS

Supplementary Materials for

Ultrafast creation of a light-induced semimetallic state in strongly excited 1T-$TiSe_2$

Maximilian Huber *et al.*

Corresponding author: Alessandra Lanzara, alanzara@lbl.gov

Sci. Adv. **10**, eadl4481 (2024)
DOI: 10.1126/sciadv.adl4481

This PDF file includes:

Supplementary Text
Figs. S1 to S7
References

Supplementary Note 1: Data Processing and Energy Alignment

All spectra in this work are taken on samples from the same batch besides the one taken at room temperature with XUV light, shown in Figure 1b. The sample on which this data is taken has a slightly higher amount of Ti self-doping and thus shows a more pronounced conduction band (*66*).

All ARPES data in this paper were analyzed using pyARPES, an open-source python-based analysis framework (*67*). Momentum distribution curves (MDCs) have been smoothed with the Gaussian method over a window of 0.0075 $Å^{-1}$(0.005 $Å^{-1}$ in Figure 1). The ARPES image plots shown in Figure 1b-e have been smoothed by 10 meV and 0.0075 $Å^{-1}$. Furthermore, for enhanced clarity we subtract an exponential background above and a constant background below the Fermi level for all image plots of excited spectra. As shown in Figure S1, the linear dispersing states above the Fermi level are already clearly visible in the raw data.

To correct for rigid band shifts due to for instance space charge effects we aligned all spectra in Figure 2 and 3 of the main text in energy to the $Se_{4p\text{-}1}$ position at the side of the band (-0.12$Å^{-1}$). Figure S2b and c show EDCs taken for different excitation densities with and without aligning, respectively. The region where the EDCs are extracted is indicated by the white line in panel a. The $Se_{4p\text{-}1}$ position is obtained by fitting the EDCs with two Voigt peaks on a linear background.

Supplementary Note 2: MDC Fitting

Figure S3 shows schematically fitted MDCs at high and low binding energies. For low binding energy the MDCs are fitted with two Lorentzian peaks with a linear background, corresponding to the two spin-orbit split selenium bands, whereas close and above the Fermi level only one peak corresponding to the $Se_{4p\text{-}1}$ band is necessary.

Supplementary Note 3: Band Overlap in the Semimetallic Phase

The light induced semimetallic state shows a pronounced indirect band overlap between valence band at *A* point and the conduction band at *L* point. Unfortunately the XUV probe energy of 22.3 eV is not high enough to fully map the *A* and *L* point simultaneously. To estimate the overlap Δ, in Figure S4 we fit the dispersion of the Ti_{3d} conduction band extracted by fitting EDCs with single Gaussian peaks on a linear background between -0.8 to -0.4$Å^{-1}$(orange markers) with a parabolic function and extrapolate it to the *L* point (solid orange line). The maximum of the valence band at *A* point is obtained by fitting the EDC again with a single Gaussian peak on a linear background and is indicated by the black line, the dispersion of the valence band is schematically shown as a guide to the eye by the solid red line. In doing so one obtains an indirect band overlap Δ between conduction band minimum at *L* point and valence band maximum at *A* point on the order of ~350 meV.

Supplementary Note 4: Polarization Dependence
Figure S5 shows a systematic comparison between different XUV probe polarizations. In the spectra taken with p-polarization the spin-orbit splitting is not well resolved and consequently the light-induced semimetallic state is barely visible.

Supplementary Note 5: Equilibrium Band Structure at High Temperatures
Figure S6 shows data taken at 300 (panel a) and 400 K (panel b) at the microARPES endstation at beamline 7.0.2 (MAESTRO) at the Advanced Light Source. Samples were measured using a Scienta R4000 Hemispherical Analyzer using circular polarized light with a photon energy of 119 eV.

Supplementary Note 6: Computational Details
First-principles calculations were performed within the density functional theory (DFT) with ultrasoft pseudopotentials (*68*), as implemented in the Quantum ESPRESSO package (*69, 70*), while employing a kinetic energy cutoff of 52 Ry for the plane-wave expansion of the Kohn-Sham wavefunctions and 575 Ry for the density. For the exchange-correlation potential we have adopted the DFT+U approximation using as semilocal DFT exchange and correlation kernel the generalized gradient approximation in the Perdew, Burke and Ernzerhof (PBE) (*71*) formulation. An on-site Hubbard U parameter equal to 3.5 eV was added to the PBE Hamiltonian, in the rotationally invariant scheme of Liechtenstein et al. (*54*). The Hubbard U value was chosen in order to best reproduce the measured electronic structure. While the local correlations included via the Hubbard U do not correctly reproduce $TiSe_2$'s dynamical instability (*23*), they still allow an excellent description of its electronic structure both for the CDW and the normal state (*23*) as long as the experimental structure is used and with a much lower computational cost with respect to hybrid approaches (*21, 72*). As our calculations are aimed to the interpretation of the experimental electronic structure, we employed the experimental structural parameters both for the normal and charge-density wave phase (*33, 73*). A 24×24×12 Monkhorst-Pack wave-vector grid (*74*) has been adopted for the integration of the Brillouin zone of the normal state unit cell, and the sampling was adapted consistently in the supercell calculations in order to maintain the same k-point density. Spin-orbit coupling was included in the calculations. Due to the semimetallic nature of the compound, a smearing approach (Methfessel-Paxton smearing (*75*) of 0.01 Ry) has been used to converge the self-consistent calculations.

Further calculations were performed employing a reduced value for the Hubbard U (2.5 eV, ≈28.6% reduction of the original value) and a reduced value for the charge-density wave distortion (0% and 50% of the initial CDW amplitude) in order to simulate the screening effects of the photoexcited carriers and the partial and total melting of the CDW phase. The theoretical calculations are performed on a 2×2×2 supercell and then unfolded to the primitive cell of $TiSe_2$ via the band unfolding method (*76*) as implemented in the BandsUP software (*77*).

By comparison with literature (*24, 34, 35*) we estimate that our XUV beam probes the k_z plane around ~ -0.33 Å^{-1}. However, due to the symmetry properties of the material there exists an indeterminacy regarding the sign of k_z when comparing it with the calculations, depending on which z-axis orientation is chosen. Thus, while we show the calculated band structure for k_z=-0.3Å^{-1} in the main text, we also report the results for k_z=0.3Å^{-1} in Figure S7 for completeness. Comparison with Figure 4 from the main text shows that there are no qualitative differences and all key results are apparent in both k_z planes.

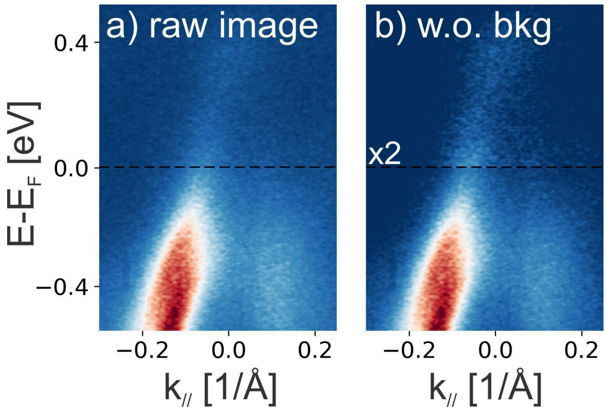

Figure S1: Raw spectra processing. a-b) Raw ARPES spectrum at a delay of 80 fs after excitation with 200 $^{\mu J}/cm^2$ before (a) and after (b) background subtraction.

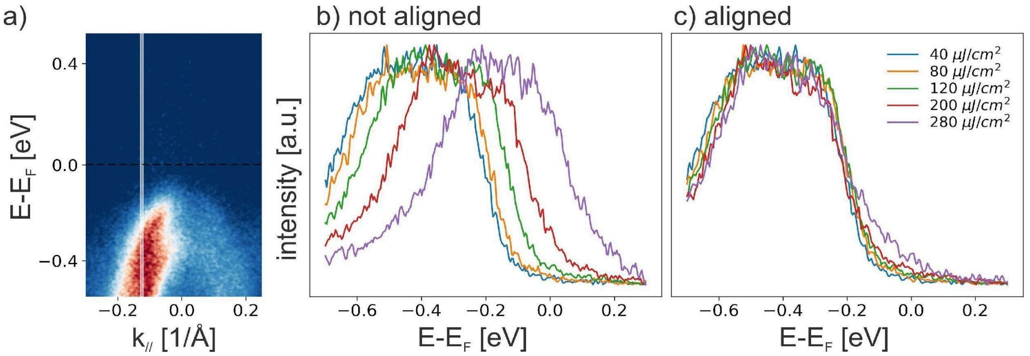

Figure S2: Energy Alignment a) ARPES plot at a delay of 85 fs after excitation with 40 $^{\mu J}/cm^2$ (spectrum identical to Figure 3a in the main text). b) EDCs extracted for different fluences. The region where the EDCs are extracted is schematically indicated by the white line in panel a. c) same EDCs as in panel b but aligned in energy.

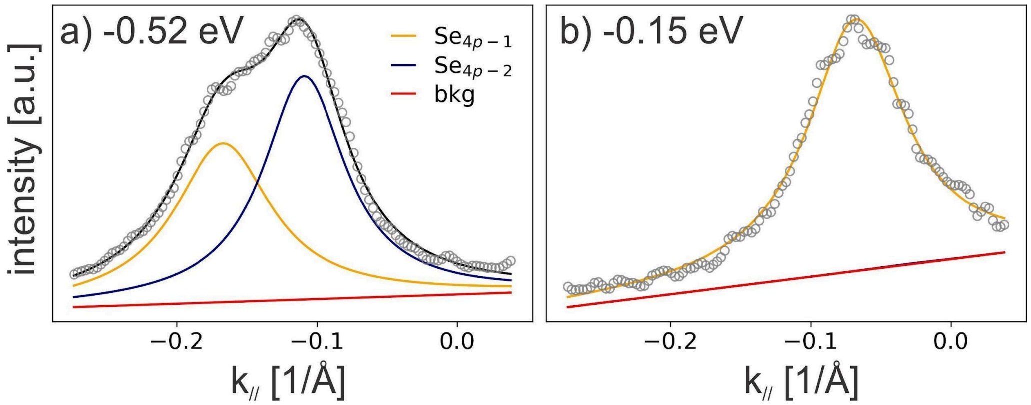

Figure S3: MDC fitting a-b) Fitted MDC taken at -0.52 eV (a) and -0.15 eV (b) for a spectrum taken at a delay of 23 fs after excitation with 180 $^{\mu J}/cm^2$.

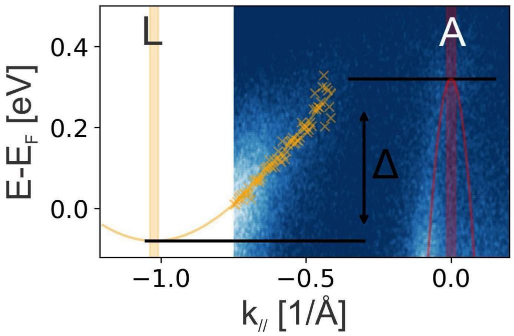

Figure S4: Estimated band overlap. ARPES image plot after excitation with 280 $^{\mu J}/cm^2$ at a delay of 85 fs. Orange crosses are fits to the Ti_{3d} conduction band. Solid orange line is a parabolic fit to the data points which is extrapolated to *L* point. Solid red line schematically shows the valence band dispersion as a guide to the eye.

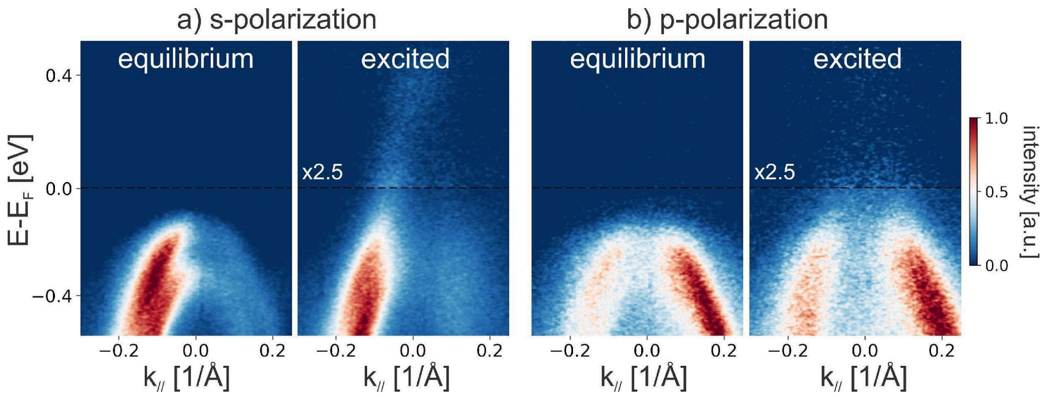

Figure S5: Polarization dependence of the XUV probe. ARPES image plots under equilibrium as well as after excitation with 200 (a) and 160 $^{\mu J}/cm^2$ (b) respectively. Spectra in panel a are taken with s-polarized, spectra in panel b with p-polarized XUV light.

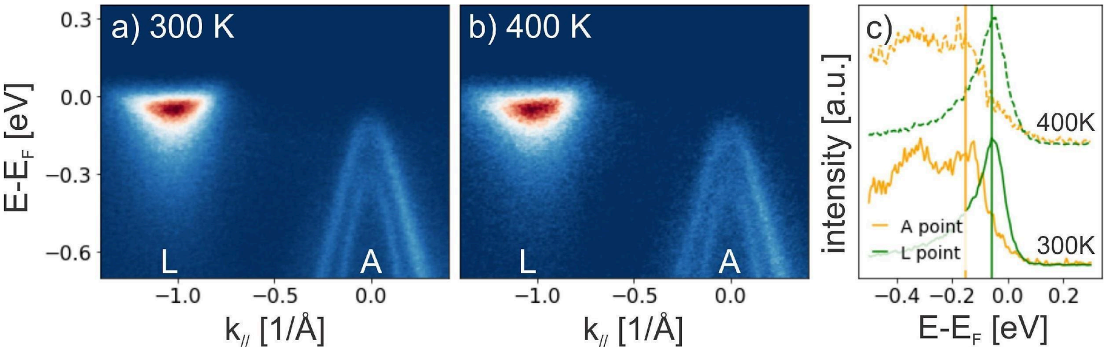

Figure S6: High temperature ARPES spectra a-b) Equilibrium ARPES spectra taken at 300 (a) and 400 K (b), respectively. Spectra were taken with 119 eV. c) Extracted EDCs from *A* (orange) and *L* (green) point for 300 (solid lines) and 400 K (dashed line), respectively.

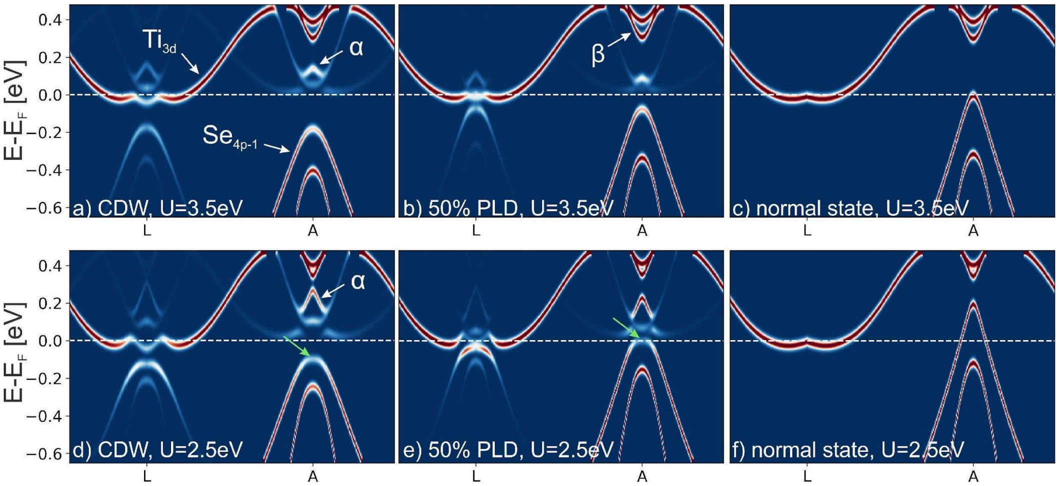

Figure S7: Calculated single-particle band structures for different lattice distortions and Hubbard U terms for k_z=0.3Å^{-1}.

REFERENCES AND NOTES

1. D. A. Siegel, C. H. Park, C. Hwang, J. Deslippe, A. V. Fedorov, S. G. Louie, A. Lanzara, Many-body interactions in quasi-freestanding graphene. *Proc. Natl. Acad. Sci. U.S.A.* **108**, 11365–11369 (2011).

2. D. A. Siegel, W. Regan, A. V. Fedorov, A. Zettl, A. Lanzara, Charge-carrier screening in single-layer graphene. *Phys. Rev. Lett.* **110**, 146802 (2013).

3. K.-F. Berggren, B. E. Sernelius, Band-gap narrowing in heavily doped many-valley semiconductors. *Phys. Rev. B* **24**, 1971–1986 (1981).

4. A. C. Riis-Jensen, J. Lu, K. S. Thygesen, Electrically controlled dielectric band gap engineering in a two-dimensional semiconductor. *Phys. Rev. B* **101**, 121110 (2020).

5. Z. R. Ye, Y. Zhang, F. Chen, M. Xu, J. Jiang, X. H. Niu, C. H. Wen, L. Y. Xing, X. C. Wang, C. Q. Jin, B. P. Xie, D. L. Feng, Extraordinary doping effects on quasiparticle scattering and bandwidth in iron-based superconductors. *Phys. Rev. X* **4**, 031041 (2014).

6. N. Dale, R. Mori, M. I. B. Utama, J. D. Denlinger, C. Stansbury, C. G. Fatuzzo, S. Zhao, K. Lee, T. Taniguchi, K. Watanabe, C. Jozwiak, A. Bostwick, E. Rotenberg, R. J. Koch, F. Wang, A. Lanzara, Correlation-driven electron-hole asymmetry in graphene field effect devices. *npj Quantum Mater.* **7**, 9 (2022).

7. D. K. Efetov, P. Kim, Controlling electron-phonon interactions in graphene at ultrahigh carrier densities. *Phys. Rev. Lett.* **105**, 256805 (2010).

8. D. C. Elias, R. V. Gorbachev, A. S. Mayorov, S. V. Morozov, A. A. Zhukov, P. Blake, L. A. Ponomarenko, I. V. Grigorieva, K. S. Novoselov, F. Guinea, A. K. Geim, Dirac cones reshaped by interaction effects in suspended graphene. *Nat. Phys.* **7**, 701–704 (2011).

9. M. M. Ugeda, A. J. Bradley, S. F. Shi, F. H. D. Jornada, Y. Zhang, D. Y. Qiu, W. Ruan, S. K. Mo, Z. Hussain, Z. X. Shen, F. Wang, S. G. Louie, M. F. Crommie, Giant bandgap renormalization and excitonic effects in a monolayer transition metal dichalcogenide semiconductor. *Nat. Mater.* **13**, 1091–1095 (2014).

10. M. Puppin, C. W. Nicholson, C. Monney, Y. Deng, R. P. Xian, J. Feldl, S. Dong, A. Dominguez, H. Hübener, A. Rubio, M. Wolf, L. Rettig, R. Ernstorfer, Excited-state band structure mapping. *Phys. Rev. B* **105**, 75417 (2022).

11. Y. Lin, Y. H. Chan, W. Lee, L. S. Lu, Z. Li, W. H. Chang, C. K. Shih, R. A. Kaindl, S. G. Louie, A. Lanzara, Exciton-driven renormalization of quasiparticle band structure in monolayer MoS2. *Phys. Rev. B* **106**, L081117 (2022).

12. C. L. Smallwood, J. P. Hinton, C. Jozwiak, W. Zhang, J. D. Koralek, H. Eisaki, D. H. Lee, J. Orenstein, A. Lanzara, Tracking Cooper pairs in a cuprate superconductor by ultrafast angle-resolved photoemission. *Science* **336**, 1137–1139 (2012).

13. C. L. Smallwood, W. Zhang, T. L. Miller, C. Jozwiak, H. Eisaki, D. H. Lee, A. Lanzara, Time- and momentum-resolved gap dynamics in Bi2Sr2CaCu2O8+δ. *Phys. Rev. B* **89**, 1–8 (2014).

14. W. Zhang, C. Hwang, C. L. Smallwood, T. L. Miller, G. Affeldt, K. Kurashima, C. Jozwiak, H. Eisaki, T. Adachi, Y. Koike, D. H. Lee, A. Lanzara, Ultrafast quenching of electron- boson interaction and superconducting gap in a cuprate superconductor. *Nat. Commun.* **5**, 1–6 (2014).

15. S. T. Ciocys, A. Lanzara, Ultrafast enhancement of electron-phonon coupling via dynamic quantum well states. *Commun. Mater.* **4**, 7 (2023).

16. A. D. L. Torre, D. M. Kennes, M. Claassen, S. Gerber, J. W. McIver, M. A. Sentef, Colloquium: Nonthermal pathways to ultrafast control in quantum materials. *Rev. Mod. Phys.* **93**, 041002 (2021).

17. L. Stojchevska, I. Vaskivskyi, T. Mertelj, P. Kusar, D. Svetin, S. Brazovskii, D. Mihailovic, Ultrafast switching to a stable hidden quantum state in an electronic crystal. *Science* **344**, 177–180 (2014).

18. A. Kogar, A. Zong, P. E. Dolgirev, X. Shen, J. Straquadine, Y. Q. Bie, X. Wang, T. Rohwer, I. C. Tung, Y. Yang, R. Li, J. Yang, S. Weathersby, S. Park, M. E. Kozina, E. J. Sie, H. Wen, P. Jarillo-Herrero, I. R. Fisher, X. Wang, N. Gedik, Light-induced charge density wave in LaTe3. *Nat. Phys.* **16**, 159–163 (2020).

19. D. Fausti, R. I. Tobey, N. Dean, S. Kaiser, A. Dienst, M. C. Hoffmann, S. Pyon, T. Takayama, H. Takagi, A. Cavalleri, Light-induced superconductivity in a stripe-ordered cuprate. *Science* **331**, 189–191 (2011).

20. S. Duan, W. Xia, C. Huang, S. Wang, L. Gu, H. Liu, D. Xiang, D. Qian, Y. Guo, W. Zhang, Ultrafast switching from the charge density wave phase to a metastable metallic state in 1T−TiSe2. *Phys. Rev. Lett.* **130**, 226501 (2023).

21. M. Hellgren, J. Baima, R. Bianco, M. Calandra, F. Mauri, L. Wirtz, Critical role of the exchange interaction for the electronic structure and charge-density-wave formation in TiSe2. *Phys. Rev. Lett.* **119**, 1–6 (2017).

22. M. Hellgren, L. Baguet, M. Calandra, F. Mauri, L. Wirtz, Electronic structure of TiSe2 from a quasi-self-consistent *G*0*W*0 approach. *Phys. Rev. B* **103**, 75101 (2021).

23. R. Bianco, M. Calandra, F. Mauri, Electronic and vibrational properties of TiSe2 in the charge-density-wave phase from first principles. *Phys. Rev. B* **92**, 1–19 (2015).

24. K. Rossnagel, L. Kipp, M. Skibowski, Charge-density-wave phase transition in1T−TiSe2: Excitonic insulator versus band-type Jahn-Teller mechanism. *Phys. Rev. B* **65**, 1–7 (2002).

25. K. Rossnagel, Suppression and emergence of charge-density waves at the surfaces of layered 1T-TiSe2 and 1T-TaS2 by in situ Rb deposition. *New J. Phys.* **12**, 125018 (2010).

26. K. Rossnagel, On the origin of charge-density waves in select layered transition-metal dichalcogenides. *J. Phys. Condens. Matter* **23**, 213001 (2011).

27. T. Rohwer, S. Hellmann, M. Wiesenmayer, C. Sohrt, A. Stange, B. Slomski, A. Carr, Y. Liu, L. M. Avila, M. Kallasignne, S. Mathias, L. Kipp, K. Rossnagel, M. Bauer, Collapse of long-range charge order tracked by time-resolved photoemission at high momenta. *Nature* **471**, 490–493 (2011).

28. M. Porer, U. Leierseder, Non-thermal separation of electronic and structural orders in a persisting charge density wave. *Nat. Mater.* **13**, 857–861 (2014).

29. A. Wegner, J. Zhao, J. Li, J. Yang, A. A. Anikin, G. Karapetrov, D. Louca, U. Chatterjee, Evidence for breathing-type pseudo Jahn-Teller distortions in the charge density wave phase of 1t−TiSe2. *Phys. Rev. B* **101**, 195145 (2020).

30. G. Gatti, A. Crepaldi, M. Puppin, N. Tancogne-Dejean, L. Xian, U. D. Giovannini, S. Roth, S. Polishchuk, P. Bugnon, A. Magrez, H. Berger, F. Frassetto, L. Poletto, L. Moreschini, S. Moser, A. Bostwick, E. Rotenberg, A. Rubio, M. Chergui, M. Grioni, Light-induced renormalization of the dirac quasiparticles in the nodal-line semimetal ZrSiSe. *Phys. Rev. Lett.* **125**, 076401 (2020).

31. A. Damascelli, Z. Hussain, Z. X. Shen, Angle-resolved photoemission studies of the cuprate superconductors. *Rev. Mod. Phys.* **75**, 473–541 (2003).

32. H. Iwasawa, High-resolution angle-resolved photoemission spectroscopy and microscopy. *Electron. Struct.* **2**, 043001 (2020).

33. F. J. Di Salvo, D. E. Moncton, J. V. Waszczak, Electronic properties and superlattice formation in the semimetal TiSe2. *Phys. Rev. B* **14**, 4321–4328 (1976).

34. M. D. Watson, O. J. Clark, F. Mazzola, I. Markovic, V. Sunko, T. K. Kim, K. Rossnagel, P. D. King, Orbital- and kz-selective hybridization of Se 4p and Ti 3d states in the charge density wave phase of TiSe2. *Phys. Rev. Lett.* **122**, 1–6 (2019).

35. P. Chen, Y. H. Chan, X. Y. Fang, S. K. Mo, Z. Hussain, A. V. Fedorov, M. Y. Chou, T. C. Chiang, Hidden order and dimensional crossover of the charge density waves in TiSe2. *Sci. Rep.* **6**, 37910 (2016).

36. C. Monney, E. F. Schwier, M. G. Garnier, N. Mariotti, C. Didiot, H. Beck, P. Aebi, H. Cercellier, J. Marcus, C. Battaglia, H. Berger, A. N. Titov, Temperature-dependent photoemission on1T-TiSe2: Interpretation within the exciton condensate phase model. *Phys. Rev. B* **81**, 1–9 (2010).

37. M. Huber, Y. Lin, N. Dale, R. Sailus, S. Tongay, R. A. Kaindl, A. Lanzara, Revealing the order parameter dynamics of 1T-TiSe2 following optical excitation. *Sci. Rep.* **12**, 15860 (2022).

38. M. Huber, Y. Lin, N. Dale, R. Sailus, S. Tongay, R. A. Kaindl, A. Lanzara, Mapping the dispersion of the occupied and unoccupied band structure in photoexcited 1T-TiSe2. *J. Phys. Chem. Solid* **168**, 110740 (2022).

39. S. Mathias, S. Eich, J. Urbancic, S. Michael, A. V. Carr, S. Emmerich, A. Stange, T. Popmintchev, T. Rohwer, M. Wiesenmayer, A. Ruffing, S. Jakobs, S. Hellmann, P. Matyba, C. Chen, L. Kipp, M. Bauer, H. C. Kapteyn, H. C. Schneider, K. Rossnagel, M. M. Murnane, M.

Aeschlimann, Self-amplified photo-induced gap quenching in a correlated electron material. *Nat. Commun.* **7**, 1–8 (2016).

40. S. Hellmann, T. Rohwer, M. Kallane, K. Hanff, C. Sohrt, A. Stange, A. Carr, M. M. Murnane, H. C. Kapteyn, L. Kipp, M. Bauer, K. Rossnagel, Time-domain classification of charge-density-wave insulators. *Nat. Commun.* **3**, 1069 (2012).

41. S. Duan, Y. Cheng, W. Xia, Y. Yang, C. Xu, F. Qi, C. Huang, T. Tang, Y. Guo, W. Luo, D. Qian, D. Xiang, J. Zhang, W. Zhang, Optical manipulation of electronic dimensionality in a quantum material. *Nature* **595**, 239–244 (2021).

42. H. Hedayat, C. J. Sayers, D. Bugini, C. Dallera, D. Wolverson, T. Batten, S. Karbassi, S. Friedemann, G. Cerullo, J. van Wezel, S. R. Clark, E. Carpene, E. Da Como, Excitonic and lattice contributions to the charge density wave in 1T−TiSe2 revealed by a phonon bottleneck. *Phys. Rev. Res.* **1**, 1–11 (2019).

43. G. Saathoff, L. Miaja-Avila, M. Aeschlimann, M. M. Murnane, H. C. Kapteyn, Laser-assisted photoemission from surfaces. *Phys. Rev. A* **77**, 113604 (2008).

44. C. L. Smallwood, W. Zhang, T. L. Miller, G. Affeldt, K. Kurashima, C. Jozwiak, T. Noji, Y. Koike, H. Eisaki, D. H. Lee, R. A. Kaindl, A. Lanzara, Influence of optically quenched superconductivity on quasiparticle relaxation rates in Bi2Sr2CaCu2O8+δ. *Phys. Rev. B* **92**, 1–6 (2015).

45. R. Mori, S. Ciocys, K. Takasan, P. Ai, K. Currier, T. Morimoto, J. E. Moore, A. Lanzara, Spin-polarized spatially indirect excitons in a topological insulator. *Nature* **614**, 249–255 (2023).

46. S. Y. Zhou, G.-H. Gweon, A. V. Fedorov, P. N. First, W. A. D. Heer, D.-H. Lee, F. Guinea, A. H. C. Neto, A. Lanzara, Substrate-induced bandgap opening in epitaxial graphene. *Nat. Mater.* **6**, 770–775 (2007).

47. S. Y. Zhou, D. A. Siegel, A. V. Fedorov, A. Lanzara, Departure from the conical dispersion in epitaxial graphene. *Physica E* **40**, 2642–2647 (2008).

48. S. Mor, M. Herzog, D. Golez, P. Werner, M. Eckstein, N. Katayama, M. Nohara, H. Takagi, T. Mizokawa, C. Monney, J. Stähler, Ultrafast electronic band gap control in an excitonic insulator. *Phys. Rev. Lett.* **119**, 1–5 (2017).

49. M. L. Mottas, T. Jaouen, B. Hildebrand, M. Rumo, F. Vanini, E. Razzoli, E. Giannini, C. Barreteau, D. R. Bowler, C. Monney, H. Beck, P. Aebi, Semimetal-to-semiconductor transition and charge-density-wave suppression in 1T−TiSe2−*x*S*x* single crystals. *Phys. Rev. B* **99**, 155103 (2019).

50. J. F. Zhao, H. W. Ou, G. Wu, B. P. Xie, Y. Zhang, D. W. Shen, J. Wei, L. X. Yang, J. K. Dong, M. Arita, H. Namatame, M. Taniguchi, X. H. Chen, D. L. Feng, Evolution of the electronic structure of 1T-Cu*x*TiSe2. *Phys. Rev. Lett.* **99**, 146401 (2007).

51. M. L. Adam, H. Zhu, Z. Liu, S. Cui, P. Zhang, Y. Liu, G. Zhang, X. Wu, Z. Sun, L. Song, Charge density wave phase suppression in 1T-TiSe2 through Sn intercalation. *Nano Res.* **15**, 2643–2649 (2022).

52. T. Jaouen, A. Pulkkinen, M. Rumo, G. Kremer, B. Salzmann, C. W. Nicholson, M. L. Mottas, E. Giannini, S. Tricot, P. Schieffer, B. Hildebrand, C. Monney, Carrier-density control of the quantum-confined1T−TiSe2 charge density wave. *Phys. Rev. Lett.* **130**, 226401 (2023).

53. G. Grüner, *Density Waves in Solids* (Perseus Publishing, ed. 1, 1994).

54. A. I. Liechtenstein, V. I. Anisimov, J. Zaanen, Density-functional theory and strong interactions: Orbital ordering in Mott-Hubbard insulators. *Phys. Rev. B* **52**, 5467–5470 (1995).

55. N. Tancogne-Dejean, M. A. Sentef, A. Rubio, Ultrafast modification of Hubbard U in a strongly correlated material: Ab initio high-harmonic generation in NiO. *Phys. Rev. Lett.* **121**, 097402 (2018).

56. N. Tancogne-Dejean, M. A. Sentef, A. Rubio, Ultrafast transient absorption spectroscopy of the charge-transfer insulator NiO: Beyond the dynamical Franz-Keldysh effect. *Phys. Rev. B* **102**, 115106 (2020).

57. D. R. Baykusheva, H. Jang, A. A. Husain, S. Lee, S. F. Tenhuisen, P. Zhou, S. Park, H. Kim, J. K. Kim, H. D. Kim, M. Kim, S. Y. Park, P. Abbamonte, B. J. Kim, G. D. Gu, Y. Wang, M. Mitrano, Ultrafast renormalization of the on-site coulomb repulsion in a cuprate superconductor. *Phys. Rev. X* **12**, 011013 (2022).

58. E. Möhr-Vorobeva, S. L. Johnson, P. Beaud, U. Staub, R. De Souza, C. Milne, G. Ingold, J. Demsar, H. Schaefer, A. Titov, Nonthermal melting of a charge density wave in TiSe2. *Phys. Rev. Lett.* **107**, 1–4 (2011).

59. D. Wegkamp, M. Herzog, L. Xian, M. Gatti, P. Cudazzo, C. L. McGahan, R. E. Marvel, R. F. Haglund, A. Rubio, M. Wolf, J. Stähler, Instantaneous band gap collapse in photoexcited monoclinic VO2 due to photocarrier doping. *Phys. Rev. Lett.* **113**, 216401 (2014).

60. J. C. Petersen, S. Kaiser, N. Dean, A. Simoncig, H. Y. Liu, A. L. Cavalieri, C. Cacho, I. C. Turcu, E. Springate, F. Frassetto, L. Poletto, S. S. Dhesi, H. Berger, A. Cavalleri, Clocking the melting transition of charge and lattice order in 1T-TaS2 with ultrafast extreme-ultraviolet angle-resolved photoemission spectroscopy. *Phys. Rev. Lett.* **107**, 1–5 (2011).

61. G. Coslovich, A. F. Kemper, S. Behl, B. Huber, H. A. Bechtel, T. Sasagawa, M. C. Martin, A. Lanzara, R. A. Kaindl, Ultrafast dynamics of vibrational symmetry breaking in a charge-ordered nickelate. *Sci. Adv.* **3**, 1–8 (2017).

62. A. Zong, A. Kogar, Y. Q. Bie, T. Rohwer, C. Lee, E. Baldini, E. Ergecen, M. B. Yilmaz, A. Freelon, E. J. Sie, H. Zhou, J. Straquadine, P. Walmsley, P. E. Dolgirev, A. V. Rozhkov, I. R. Fisher, P. Jarillo-Herrero, B. V. Fine, N. Gedik, Evidence for topological defects in a photoinduced phase transition. *Nat. Phys.* **15**, 27–31 (2019).

63. M. R. Otto, J.-H. Poehls, L. P. Rene de Cotret, M. J. Stern, M. Sutton, B. J. Siwick, Mechanisms of electron-phonon coupling unraveled in momentum and time: The case of soft phonons in TiSe2. *Nat. Commun.* **7**, eabf2810 (2021).

64. T. Heinrich, H.-T. Chang, S. Zayko, K. Rossnagel, M. Sivis, C. Ropers, Electronic and structural fingerprints of charge-density-wave excitations in extreme ultraviolet transient absorption spectroscopy. *Phys. Rev. X* **13**, 021033 (2023).

65. J. H. Buss, H. Wang, Y. Xu, J. Maklar, F. Joucken, L. Zeng, S. Stoll, C. Jozwiak, J. Pepper, Y. D. Chuang, J. D. Denlinger, Z. Hussain, A. Lanzara, R. A. Kaindl, A setup for extreme-ultraviolet ultrafast angle-resolved photoelectron spectroscopy at 50-kHz repetition rate. *Rev. Sci. Instrum.* **90**, 023105 (2019).

66. T. Jaouen, B. Hildebrand, M. L. Mottas, M. Di Giovannantonio, P. Ruffieux, M. Rumo, C. W. Nicholson, E. Razzoli, C. Barreteau, A. Ubaldini, E. Giannini, F. Vanini, H. Beck, C. Monney, P. Aebi, Phase separation in the vicinity of Fermi surface hot spots. *Phys. Rev. B* **100**, 1–11 (2019).

67. C. Stansbury, A. Lanzara, Pyarpes: An analysis framework for multimodal angle-resolved photoemission spectroscopies. *SoftwareX* **11**, 100472 (2020).

68. D. Vanderbilt, Soft self-consistent pseudopotentials in a generalized eigenvalue formalism. *Phys. Rev. B* **41**, 7892–7895 (1990).

69. P. Giannozzi, S. Baroni, N. Bonini, M. Calandra, R. Car, C. Cavazzoni, D. Ceresoli, G. L. Chiarotti, M. Cococcioni, I. Dabo, A. D. Corso, S. de Gironcoli, S. Fabris, G. Fratesi, R. Gebauer, U. Gerstmann, C. Gougoussis, A. Kokalj, M. Lazzeri, L. Martin-Samos, N. Marzari, F. Mauri, R. Mazzarello, S. Paolini, A. Pasquarello, L. Paulatto, C. Sbraccia, S. Scandolo, G. Sclauzero, A. P. Seitsonen, A. Smogunov, P. Umari, R. M. Wentzcovitch, QUANTUM ESPRESSO: A modular and open-source software project for quantum simulations of materials. *J. Phys. Condens. Matter* **21**, 395502 (2009).

70. P. Giannozzi, O. Baseggio, P. Bonfa, D. Brunato, R. Car, I. Carnimeo, C. Cavazzoni, S. de Gironcoli, P. Delugas, F. Ferrari Ruffino, A. Ferretti, N. Marzari, I. Timrov, A. Urru, S. Baroni, Quantum ESPRESSO toward the exascale. *J. Chem. Phys.* **152**, 154105 (2020).

71. J. P. Perdew, K. Burke, M. Ernzerhof, Generalized gradient approximation made simple. *Phys. Rev. Lett.* **77**, 3865–3868 (1996).

72. J. Heyd, G. E. Scuseria, M. Ernzerhof, Hybrid functionals based on a screened Coulomb potential. *J. Chem. Phys.* **118**, 8207–8215 (2003).

73. F. Weber, S. Rosenkranz, J.-P. Castellan, R. Osborn, G. Karapetrov, R. Hott, R. Heid, K.-P. Bohnen, A. Alatas, Electron-phonon coupling and the soft phonon mode in Tise2. *Phys. Rev. Lett.* **107**, 266401 (2011).

74. H. J. Monkhorst, J. D. Pack, Special points for brillouin-zone integrations. *Phys. Rev. B* **13**, 5188–5192 (1976).

75. M. Methfessel, A. T. Paxton, High-precision sampling for brillouin-zone integration in metals. *Phys. Rev. B* **40**, 3616–3621 (1989).

76. V. Popescu, A. Zunger, Extracting E versus $\vec{k}$ effective band structure from supercell calculations on alloys and impurities. *Phys. Rev. B* **85**, 085201 (2012).

77. P. V. C. Medeiros, S. Stafstrom, J. Bjork, Effects of extrinsic and intrinsic perturbations on the electronic structure of graphene: Retaining an effective primitive cell band structure by band unfolding. *Phys. Rev. B* **89**, 041407 (2014).
